# Streamlining performance prediction: data-driven KPIs in all swimming strokes

**DOI:** 10.1186/s13104-024-06714-x

**Published:** 2024-02-19

**Authors:** Craig A. Staunton, Michael Romann, Glenn Björklund, Dennis-Peter Born

**Affiliations:** 1 Swedish Winter Sports Research Centre, Department of Health Sciences, Mid Sweden University, Östersund, Sweden; 2 Department for Elite Sport, Swiss Federal Institute of Sport, Hochschule Lärchenplatz, 2532 Magglingen, Switzerland; 3 Section for High-Performance Sports, Swiss Swimming Federation, Bern, Switzerland

**Keywords:** Competitive swimming, Data analysis, Key performance indicators, Performance prediction, Training strategies

## Abstract

**Objective:**

This study aimed to identify Key Performance Indicators (KPIs) for men’s swimming strokes using Principal Component Analysis (PCA) and Multiple Regression Analysis to enhance training strategies and performance optimization. The analyses included all men’s individual 100 m races of the 2019 European Short-Course Swimming Championships.

**Results:**

Duration from 5 m prior to wall contact (In5) emerged as a consistent KPI for all strokes. Free Swimming Speed (FSS) was identified as a KPI for 'continuous' strokes (Breaststroke and Butterfly), while duration from wall contact to 10 m after (Out10) was a crucial KPI for strokes with touch turns (Breaststroke and Butterfly). The regression model accurately predicted swim times, demonstrating strong agreement with actual performance. Bland and Altman analyses revealed negligible mean biases: Backstroke (0% bias, LOAs − 2.3% to + 2.3%), Breaststroke (0% bias, LOAs − 0.9% to + 0.9%), Butterfly (0% bias, LOAs − 1.2% to + 1.2%), and Freestyle (0% bias, LOAs − 3.1% to + 3.1%). This study emphasizes the importance of swift turning and maintaining consistent speed, offering valuable insights for coaches and athletes to optimize training and set performance goals. The regression model and predictor tool provide a data-driven approach to enhance swim training and competition across different strokes.

**Supplementary Information:**

The online version contains supplementary material available at 10.1186/s13104-024-06714-x.

## Introduction

Competitive swimming encompasses four primary techniques: the front crawl or freestyle (FR), breaststroke (BR), backstroke (BA), and the butterfly (BU). Swimmers often specialize in specific strokes or distances, showcasing their expertise in the water [1]. Identifying Key Performance Indicators (KPIs) for each stroke becomes crucial for coaches and athletes to guide training strategies and optimize performance [2].

It's evident that KPIs can vary significantly between strokes, given the distinct characteristics and techniques involved. For example, prior research has revealed different key somatic features of the 4 swimming strokes [3, 4]. Further, strokes with alternating arm movements, like freestyle and backstroke, may have different KPIs compared to those with continuous stroke actions, such as the butterfly and breaststroke [5]. Additionally, the nuances of turning, (i.e. tumble turn for alternating and touch turn for continuous swimming strokes), play a substantial role in influencing KPIs across different strokes [6].

With the ever-evolving landscape of competitive swimming and interdisciplinary experts involved in the support system, a wealth of performance data accompanies both training and competitions [7]. As advancements in technology continue to provide more sophisticated race analysis and greater accessibility to performance data, the challenges of managing 'big data' in this field are growing. Despite this, some more recent research has used advanced statistical techniques in order to model swimming performance [8–10]. Furthermore, it is foreseeable that the future will bring increased prevalence of automated tracking systems and motion sensors integrated with swimmers. However, sifting through these data to discern its significance can be challenging for coaches and athletes. Data reduction techniques, such as Principal Component Analyses (PCA), provide a valuable means of extracting essential information that explain the most significant variances in performance and eliminate redundant variables that capture similar information (for more information about PCA please see the following reviews [11, 12]). For example, PCA has been utilised previously within sports such as swimming [13], skeleton [14] or rugby [15] to help with data reduction. When complemented by Multiple Regression Analysis, these techniques enable the identification and comparison of KPIs specific to each stroke.

With these complexities in mind, this study's primary objective is to explore the nuances of men’s swimming strokes. By employing data reduction techniques like PCA and Multiple Regression Analysis, we aim to achieve two key goals. Firstly, we seek to uncover KPIs across the four swimming strokes, offering deeper insights into each stroke's unique intricacies. Secondly, our study aims to develop a performance prediction tool that can be used practically by coaches and athletes to monitor performance.

## Material and methods

### Participants

Participants included all men’s individual 100 m races of the 2019 European Short-Course Swimming Championships in Glasgow, Scotland. Races included the FR, BA, BR and BU (FR: N = 74; swimming points = 782 ± 79; BA: N = 62; swimming points = 801 ± 84; BR: N = 47; swimming points = 826 ± 82; BU: N = 61; swimming points = 775 ± 78). All swimmers that participate at events hosted by the European Swimming Association LEN (Ligue Européenne de Natation) agree to be video monitored for television broadcasting and race analysis of the participating nations. The study was pre-approved by the leading institution’s internal review board (registration number: 098-LSP-191119) and was in accordance to the latest version of the code of conduct of the World Medical Association for studies involving human subjects (Helsinki Declaration).

### Data collection

A twelve-camera system (Spiideo, Malmö, Sweden) was employed to monitor all races. Ten cameras followed each individual swimmer and two fixed-view cameras monitored the start and turn sections of all swimmers. Split times, stroke rate (SR), distance per stroke (DPS), and the duration from the starting beep to the head passing the 5 m, 10 m, and 15 m marks (start5, start10, start15) were post processed by manual digitalization by a single assessor who was an expert race analyst (Kinovea 0.9.1; Joan Charmant & Contrib., https://kinovea.org/). Similarly, the duration from 5 m prior to the moment of wall contact (in5), the duration from the wall contact to the head passing the 5 m after the turn (out5), and the duration from the wall contact to the head passing the 10 m after the turn (out10) was determined for every turn. Free-swimming speed (FSS) was calculated from the middle 10 m section of each lap from the difference between split time, out10 and in5. FSS was not calculated for the first lap given the influence of the start on swimming speed. The average of each metric was calculated across all laps for each race. Reliability of the data analysis has previously been determined with an intra-class correlation coefficient of 0.98 ± 0.04 [16–18].

### Development of the potential predictor

A practical tool was developed using Microsoft Excel (Additional file 1) further referred to as the Potential Predictor. The Potential Predictor was designed to utilise the identified KPIs for each stroke type allowing coaches to estimate race performance times using KPIs and compare athlete performances against predicted outcomes based on these thresholds. Race times were categorised into distinct classifications based on performance outcomes: Did Not Qualify (DNQ): swimmers who did not progress beyond the heats and did not qualify for any further rounds; Qualified (Q): swimmers who successfully qualified for either the semi-final (QSF) or the final (QF); Medallists (M): swimmers who achieved podium positions and won medals in their respective events. Mean swimming time and KPIs for the performance classifications for all stroke types are displayed in Additional file 2: Table S1. To use this tool effectively, coaches should carefully consider the specific KPIs associated with each stroke type. These KPIs should be collected under optimal conditions, such as selecting the best results from multiple trials. Subsequently, these gathered KPIs can be entered into the Potential Predictor to ascertain an individual swimmer’s potential along with the lower and upper 95% LOA. Coaches can manipulate one or more KPIs to assess their impact on future race outcomes.

### Statistical analyses

To assess variables with a high degree of covariance (≥ 0.8), a covariance matrix was computed for all z-scored data. A Principal Component Analysis (PCA) was conducted on all variables with high covariances. The Kaiser–Meyer–Olkin measure was used to verify the sampling adequacy of the data, with a value of 0.5 used as a threshold for acceptability [19]. The Bartlett test of sphericity was also used to determine the suitability of the data for PCA, with significance accepted at an α level of *P* ≤ 0.05. Principal Components (PCs) with Eigenvalues greater than 1 were extracted. Orthogonal rotation (varimax) was used to improve the identification and interpretation of factors [20]. The most heavily loaded (most strongly related) variable to each component were then retained, along with the original variables which did not display a high degree of covariance, to be used as predictors for swim time (criterion) in a stepwise multiple linear regression analysis. Entered variables remained in the model if a significant R^2^ change (*P* < 0.05) was reported and the unstandardized *β* coefficients were used to form the prediction equations. The agreement between the predicted and actual swimming performances, along with the 95% limits of agreement (LOA), were subsequently analysed using methods described by Bland and Altman [21]. All statistical analyses were performed using SPSS Statistics (Version 29; IBM Corporation, NY).

## Results

PCA revealed two PCs with Eigen values > 1 for all swimming strokes. The variables which had the highest component loadings to each PC are displayed in Table 1. PC1 was most strongly correlated with Start15 for the Freestyle, Start10 for the Backstroke and Out10 for both the Breaststroke and Butterfly. PC2 was most strongly correlated with SR from the Freestyle, Backstroke and Butterfly and with Start10 for the Breaststroke.

**Table 1:** The two principal components (PCs) of the Varimax rotated component matrix for all swimming strokes and their explained variance

	FR		BA		BR		BU	
	PC 1	PC 2	PC 1	PC 2	PC 1	PC 2	PC 1	PC 2
Variance explained (%)	63.7	82.5	65.8	84.9	47.5	71.4	65.6	83.5
DPS		− 0.931		− 0.962				− 0.876
SR		**0.982**		**0.964**				**0.973**
FSS	− 0.867		− 0.841		***	***	***	***
Start5	***	***	***	***			***	***
Start5to10	0.863		0.915			0.781	0.863	
Start10	0.894		**0.926**			**0.819**	0.859	
Start10to15	0.806		0.87			0.801	0.851	
Start15	**0.956**		0.969		0.866		0.93	
In5	***	***	***	***	***	***	***	***
Out5	0.832		0.791		0.87		0.701	
Out5to10	0.849		0.926		0.875		0.891	
Out10	0.935		0.966		**0.952**		**0.936**	

Cells marked with *** represent variables that did not have high covariances and therefore were not included in the PCA, but were included in the subsequent stepwise linear regression. For better visualisation, only component loadings ≤ − 0.7 or ≥ 0.7 are displayed. Bold figures indicate the strongest component loading (those with the highest correlation to the principal component) and the metric that was retained to be used within the subsequent stepwise linear regression. *FR* Freestyle, *BA* Backstroke; *BR* Breaststroke, BU Butterfly

Stepwise multiple linear regressions revealed the KPIs for each stroke type. The unstandardized *β* coefficients were then used to form the following regression equations:

$$SwimTime\_FR = 12.194 + 4.633*Start15 + 3.330*in5$$

$$SwimTime\_BA = 4.997 + 3.416*Start15 + 8.337*in5$$

$$SwimTime\_BR = 40.415 + 4.671*in5 + 4.372*out10 {-} 13.346*FSS$$

$$SwimTime\_BU = 30.948 + 5.050*in5 + 4.358*out10 {-} 8.358*FSS$$

**Fig. 1:**
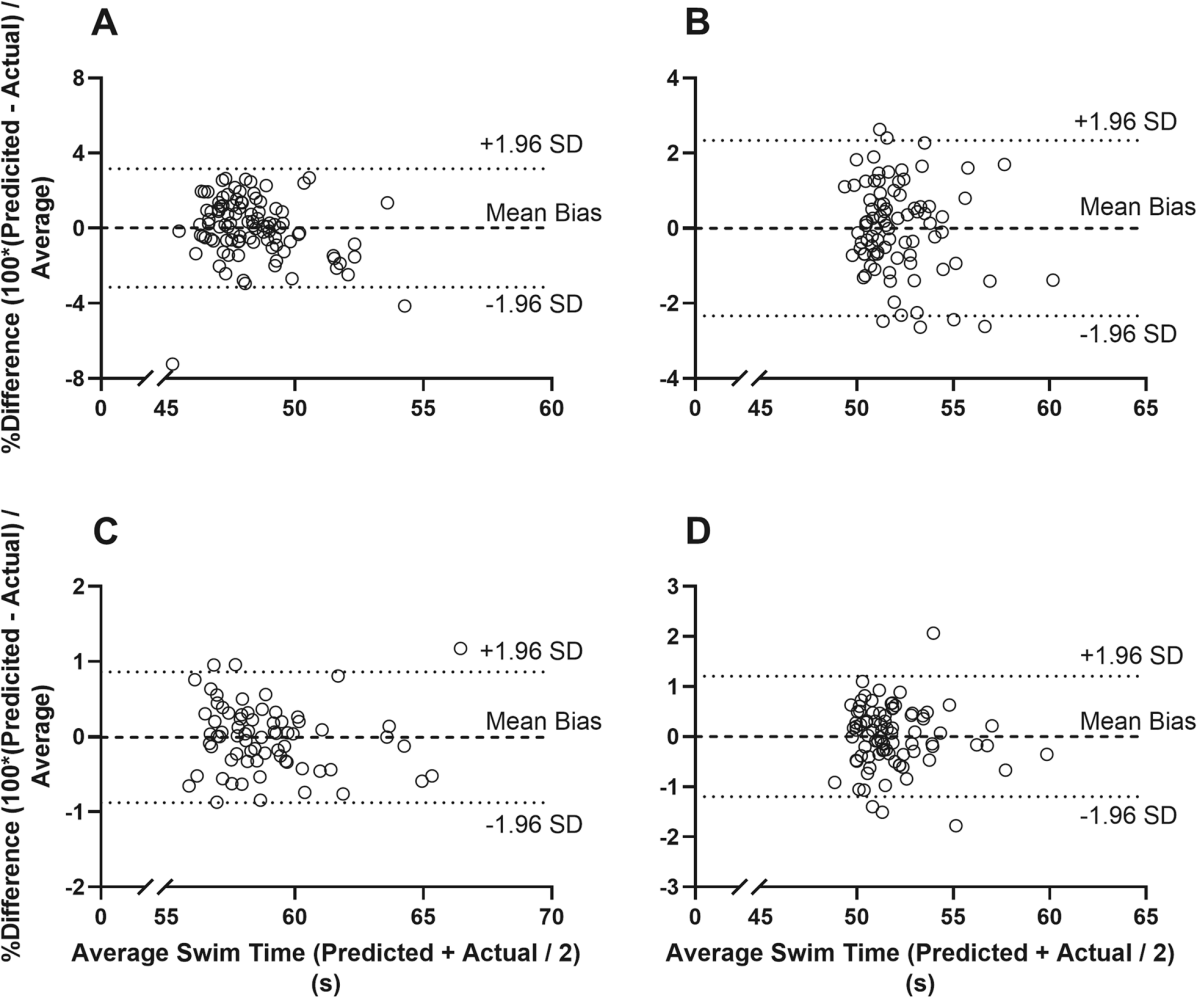
Bland and Altman plots with 95% limits of agreement displaying the agreement between predicted and actual swim time for the Freestyle (Panel** A**), Backstroke (Panel** B**), Breaststroke (Panel** C**) and Butterfly (Panel** D**) freestyle races

The results Bland and Altman plots indicate a consistently very strong agreement between predicted and actual swimming performance for all strokes, with a mean bias of 0% (Fig. 1). Specifically, for BA, the mean bias was -0.001% with 95%LOAs from − 2.3 to + 2.3% (or −1.2 to + 1.2 s; Fig. 1A). For BR, the mean bias was -0.001% with 95% LOAs from − 0.9 to + 9.9% (or − 0.5 to + 0.5 s; Fig. 1B). For BU, the mean bias was 0.003% with 95% LOAs from − 1.2 to + 1.2% (or − 0.6 to + 0.6 s; Fig. 1C) and for FR, the mean bias was 0.02% with 95% LOAs from − 3.1 to + 3.1% (or − 1.5 to + 1.5 s; Fig. 1D).

## Discussion

This study sought to uncover KPIs across various swimming strokes using data reduction techniques and multiple regression. The main findings of this study were: (1) in5 was identified as a KPI for all strokes; (2) FSS was a KPI for the ‘continuous’ swimming strokes (Breaststroke & Butterfly) but not for the ‘alternating’ strokes (Freestyle & Backstroke); (3) Out10 was identified as a KPI for the strokes involving a touch turn (Breaststroke and the Butterfly); and (4) the regression model provides a reliable method to predict swim time based on the underlying KPIs.

One of the central findings of this research is the consistent identification of in5 as a KPI for all four swimming strokes. The last 5 m leading up to the wall (in5) are intrinsically linked to FSS and holds particular significance in Freestyle and Backstroke, where in5 encapsulates the critical aspects of the tumble turn. Precisely timing the initiation and optimizing rotation velocity within these last 5 m significantly influences the outcome of in5 [22]. As such, these findings underscore the critical role of swift swimming speeds for all swimming strokes, but also efficient timing of tumble turns for the Freestyle and Backstroke for optimising performance. These findings extend prior research that has demonstrated the importance of fast turning for optimal performance in short-course swimming [16–18]. Although, swimmers perform numerous turns during their daily training routine [23], coaches should place particular attention to race pace specific turns in order to optimize timing during the wall approach.

While in5 is associated with FSS as swimmers approach the pool wall, it’s noteworthy that for 'continuous' swimming strokes like Breaststroke and Butterfly, FSS, alongside in5, emerged as a significant KPI. This finding underscores the difference in KPIs between 'continuous' swimming strokes (Breaststroke and Butterfly) and 'alternating' strokes (Freestyle and Backstroke). In essence, it suggests that these variations in KPIs align with the inherent differences in these distinct swimming techniques. Recognizing FSS as a KPI for continuous swimming strokes is consistent with earlier research showing the impact of intra-cyclic variation in horizontal velocity on overall swimming speed [5]. These findings collectively emphasize the importance of maintaining consistent speed and minimizing 'breaking forces,' especially in Breaststroke and Butterfly. In contrast, Freestyle and Backstroke generally exhibit lower intra-cyclic variation in horizontal velocity [5], potentially making FSS less distinguishing for overall swimming performance, at least in the 100 m event.

In strokes involving a touch turn, namely Breaststroke and Butterfly, our analysis has identified Out10 as a KPI. This further underscores the vital role of quick and efficient turning in optimizing performance for short-course swimming. Specifically, in the context of touch turns, Out10 encompasses a 180-degree body rotation following the initial wall contact. Furthermore, the recognition of Out10 as a KPI underscores the importance of a powerful push-off from the wall during the turn. Past research has already established the significance of tailored strength and conditioning programs on land to enhance the push-off from the pool wall and gain a competitive advantage [24]. Moreover, mastering undulating kicking is a crucial skill for preserving maximum velocity from the push-off during the underwater phase [25]. Coaches and athletes can leverage this knowledge to refine training strategies and technique development, ultimately paving the way for enhanced performance.

The regression model effectively predicts swim times based on identified KPIs, aiding coaches and athletes in informed decision-making, goal setting, and personalized training plans. Incorporating individual performance data into the model offers insights into factors influencing swim times and rankings. The 95% Limits of Agreement (LOAs) define performance range, guiding the understanding of prediction variability. Coaches and athletes must consider these LOAs to assess acceptable variability. It’s notable that the freestyle race has wider LOAs, signifying lower prediction accuracy. This information empowers coaches and athletes to make informed decisions and adjustments in their training approaches, especially in cases where predictive certainty may be lower, such as in freestyle races.

In conclusion, this study has unveiled essential insights into the performance determinants for men's swimming strokes, revealing the unique intricacies of each stroke and identifying specific KPIs. Specifically, the study highlights the importance of swift turning across all strokes and minimising speed variations and swimming efficiency, in particular for continuous swimming strokes, as well as a powerful push from the wall when turning. The regression model and predictor tool empower coaches and swimmers with the knowledge of KPIs and the ability to predict 100 m race times across different strokes.

## Limitations

- The KPIs identified in this study are based solely on their statistical significance using the specific statistical methods employed in this study.
- This does not imply that other metrics or variables are insignificant in achieving successful performance.
- A holistic approach still considers multiple factors for comprehensive evaluation.
- KPIs cannot be assessed independently. Larger effort put into one race section may interfere with performance in another phase of the race.
- The data set and predictor tool only provide data for short-course races and should be expanded to long-course races.

### Supplementary Information

**Additional file 1.** Performance Predictor Tool.

**Additional file 2.** Mean swimming time and KPIs for the performance classifications for all swimming strokes.

## Data Availability

Data are available on request by the corresponding author.

## Abbreviations

BA: Backstroke
BR: Breaststroke
BU: Butterfly
DPS: Distance per stroke
FR: Freestyle
FSS: Free Swimming Speed
In5: The duration from 5 m prior to the moment of wall contact
KPIs: Key performance indicators
LOA: Limits of agreement
PCA: Principal component analysis
Out5: The duration from the wall contact to the head passing the 5 m after the turn
Out10: The duration from the wall contact to the head passing the 10 m after the turn
SR: Stroke Rate
Start5: The duration from the starting beep to the head passing the 5 m mark
Start10: The duration from the starting beep to the head passing the 10 m mark

## References

[1] Stewart AM, Hopkins WG (2000). Consistency of swimming performance within and between competitions. Med Sci Sports Exerc.

[2] Arellano R, Ruiz-Navarro JJ, Barbosa TM, López-Contreras G, Morales-Ortíz E, Gay A (2022). Are the 50 m race segments changed from heats to finals at the 2021 European swimming championships?. Front Physiol.

[3] Nevill AM, Negra Y, Myers TD, Sammoud S, Chaabene H (2020). Key somatic variables associated with, and differences between the 4 swimming strokes. J Sports Sci.

[4] Rejman M, Nevill AM, Garrido ND, Rudnik D, Morais JE (2023). Identification of key somatic features that are common and the ones that differ between swim strokes through allometric modeling. Front Sports Active Living.

[5] Barbosa TM, Morouço P, Jesus S, Feitosa WG, Costa MJ, Marinho D (2012). The interaction between intra-cyclic variation of the velocity and mean swimming velocity in young competitive swimmers. Int J Sports Med.

[6] Cuenca-Fernández F, Ruiz-Navarro JJ, Polach M, Arellano R, Born D-P (2023). Short-course performance variation across all race sections: How 100 and 200 m elite male swimmers progress between rounds. Front Sports Active Living.

[7] Barbosa TM, Barbosa AC, Simbaña Escobar D, Mullen GJ, Cossor JM, Hodierne R (2021). The role of the biomechanics analyst in swimming training and competition analysis. Sports Biomech.

[8] Gourgoulis V, Nikodelis T (2022). Comparison of the arm-stroke kinematics between maximal and sub-maximal breaststroke swimming using discrete data and time series analysis. J Biomech.

[9] Morais JE, Marinho DA, Cobley S, Barbosa TM (2023). Identifying differences in swimming speed fluctuation in age-group swimmers by statistical parametric mapping: a biomechanical assessment for performance development. J Sports Sci Med.

[10] Morais JE, Barbosa TM, Lopes T, Moriyama S-I, Marinho DA (2023). Comparison of swimming velocity between age-group swimmers through discrete variables and continuous variables by statistical parametric mapping. Sports Biomech.

[11] Rojas-Valverde D, Pino-Ortega J, Gómez-Carmona CD, Rico-González M (2020). A systematic review of methods and criteria standard proposal for the use of principal component analysis in team’s sports science. Int J Environ Res Public Health.

[12] O’Donoghue P (2008). Principal components analysis in the selection of key performance indicators in sport. Int J Perf Anal Spor.

[13] Burkhardt D, Born D-P, Singh NB, Oberhofer K, Carradori S, Sinistaj S (2023). Key performance indicators and leg positioning for the kick-start in competitive swimmers. Sports Biomech.

[14] Colyer SL, Stokes KA, Bilzon JL, Cardinale M, Salo AI (2017). Physical predictors of elite skeleton start performance. Int J Sports Physiol Perform.

[15] Parmar N, James N, Hearne G, Jones B (2018). Using principal component analysis to develop performance indicators in professional rugby league. Int J Perf Anal Spor.

[16] Born D-P, Kuger J, Polach M, Romann M (2021). Start and turn performances of elite male swimmers: benchmarks and underlying mechanisms. Sports Biomech..

[17] Born D-P, Romann M, Stöggl T (2022). Start fast, swim faster, turn fastest: section analyses and normative data for individual medley. J Sports Sci Med.

[18] Born D-P, Kuger J, Polach M, Romann M (2021). Turn fast and win: the importance of acyclic phases in top-elite female swimmers. Sports.

[19] Kaiser HF (1974). An index of factorial simplicity. Psychometrika.

[20] Hair JF, Anderson RE, Babin BJ, Black WC (2010). Multivariate data analysis: A global perspective.

[21] Bland JM, Altman DG (1999). Measuring agreement in method comparison studies. Stat Methods Med Res.

[22] David S, Grove T, Mv D, Koster P, Beek PJ (2022). Improving tumble turn performance in swimming—the impact of wall contact time and tuck index. Front Sports Active Living.

[23] Pollock S, Gaoua N, Johnston MJ, Cooke K, Girard O, Mileva KN (2019). Training regimes and recovery monitoring practices of elite British swimmers. J Sports Sci Med.

[24] Crowley E, Harrison AJ, Lyons M (2018). Dry-land resistance training practices of elite swimming strength and conditioning coaches. J Strength Cond Res.

[25] Ruiz-Navarro JJ, Cuenca-Fernández F, Sanders R, Arellano R (2022). The determinant factors of undulatory underwater swimming performance: a systematic review. J Sports Sci.

