# Supplementary material for: Streamlining performance prediction: data-driven KPIs in all swimming strokes
Source: BMC Res Notes. 2024 Feb 19;17:52. doi: 10.1186/s13104-024-06714-x (PMC10877752; doi:10.1186/s13104-024-06714-x)
Supplement: Supplementary file 2 — Additional file 2. Mean swimming time and KPIs for the performance classifications for all swimming strokes. [file 13104_2024_6714_MOESM2_ESM.docx]

|  | FR | | | |  | BA | | | |  | BR | | | |  | BU | | | |  | |
| --- | --- | --- | --- | --- | --- | --- | --- | --- | --- | --- | --- | --- | --- | --- | --- | --- | --- | --- | --- | --- | --- |
|  | DNQ | QSF | QF | M |  | DNQ | QSF | QF | M |  | DNQ | QSF | QF | M |  | DNQ | QSF | QF | M | |  |
| SwimTime (s) | 49.4 | 47.1 | 46.8 | 45.9 |  | 53.4 | 50.9 | 50.3 | 49.8 |  | 60.7 | 57.9 | 56.8 | 56.3 |  | 53.0 | 50.7 | 50.1 | 49.4 | |  |
| FSS (m·s^-1^) |  |  |  |  |  |  |  |  |  |  | 1.49 | 1.54 | 1.57 | 1.60 |  | 1.77 | 1.84 | 1.87 | 1.87 | |  |
| In5 (s) | 2.83 | 2.64 | 2.56 | 2.64 |  | 3.08 | 2.96 | 2.96 | 2.89 |  |  |  |  |  |  | 2.64 | 2.54 | 2.48 | 2.42 | |  |
| Start15 (s) | 5.95 | 5.67 | 5.61 | 4.57 |  | 6.62 | 6.20 | 6.14 | 6.05 |  |  |  |  |  |  |  |  |  |  | |  |
| Out10 (s) |  |  |  |  |  |  |  |  |  |  | 5.98 | 5.63 | 5.56 | 5.52 |  | 5.40 | 5.15 | 5.11 | 5.02 | |  |

**Supplementary Table 1.** Mean swimming time and KPIs for the performance classifications for all swimming strokes.
